# The Chemists' Exhibition, 1900

**Published:** 1900-06-30

**Authors:** 


### THE CHEMISTS' EXHIBITION, 1900.

This annual exhibition, which was held last week at St. James's Hall, Manchester, being the sixth of the series, was very successful. For the most part it appealed to chemists, but there was a considerable attendance of medical men and nurses, who, from the marked attention devoted to the stalls, evidently found much to interest and inform them. Prominent among the array of drugs and pharmaceutical preparations were the stalls of Messrs. Oppenheimer, Sons, and Co. (Limited), with Palatinoids and Bi-Palatinoids (their Bi Palatinoid of Easton's Syrup and Creosote being noteworthy); Messrs. Parke, Davis, and Co., where Euthymol Toothpaste and Johnston's Ethereal Antiseptic Soap were specially noticed; Messrs. Woolley, Sons, and Co. (Limited), Manchester, who had a fine display, including syrup of glycero phosphates, and Akolethe (a solution of the sedative principles of opium); the Bayer Company (Limited), foremost among their preparations being Trional (a hypnotic), Aspirin (acetic ether of salicylic acid), and Europhen (odourless substitute for iodoform); Messrs. Wyleys, Coventry, their non-alcoholic tinctures and friction cakes (liniments) being particularly observed; and Messrs. Evans, Sons, and Co., Liverpool, with a handsome display of their fine preparations. Foods were represented by the British Somatose Company (Limited), with Somatose in three forms; Messrs. Brand and Co. (Limited), with meat essences, and Fever Food (essence of beef with cream and yolk of egg); Worth's Food Syndicate (Limited), with Cheltine foods; Mellins Food (Limited), with infants' and invalids' food; Peptine Maltine (Limited), Leicester, with various malted foods, and also concentrated peptonised malt tablets; and the Plasmon Syndicate (Limited), with Plasmon and Plasmon Beef Extract. The Anglo-Swiss Milk Company, in their exhibit, showed "Ideal Milk" (sterilised milk with 20 per cent. of cream). Surgical and medical bandages and plasters, appliances, &c., were shown by Messrs. A. de St. Dalmas and Co., Leicester; Messrs. J. Quilliam and Co., Manchester; W. Mather (Limited), Manchester, with rubber adhesive plasters; and Messrs. Heath Bros., Manchester. Messrs. John Weiss and Son (Limited), London, had a very fine display of surgical instruments. Mineral waters were exhibited by Messrs. Ingram and Royle (Limited), and Camwal (Limited). Messrs. Farrow and Jackson exhibited mineral water machines, which would effect much economy at institutions. The stall of the Vinolia Company (Limited), with toilet soaps and requisites, proved very attractive, the Baby Soap and Vinolia Cream receiving special attention from nurses. The stalls of many other exhibitors were of the highest interest, though space will not permit of their being particularised, and the exhibition, as a whole, constituted a most extensive and effective display.

